# Kyphoscoliotic Ehlers–Danlos syndrome associated with superior mesenteric artery aneurysm and abdominal aortic rupture: a case report

**DOI:** 10.3389/fped.2025.1737724

**Published:** 2026-01-14

**Authors:** Jiru Li, Keqiang Liu, Xiaodong Zhu, Yaya Xu, Lili Xu, Runmin Chi, Yueniu Zhu

**Affiliations:** 1 Department of Pediatric Critical Care Medicine, Xinhua Hospital, Affiliated to Shanghai Jiaotong University School of Medicine, Shanghai, China; 2 Shanghai Institute for Pediatric Research, Shanghai, China; 3 Department of Radiology, Xinhua Hospital, Affiliated to Shanghai Jiaotong University School of Medicine, Shanghai, China

**Keywords:** kyphoscoliotic Ehlers–Danlos syndrome, *PLOD*1 gene, genetic variants, therapy, variants, vascular aneurysms

## Abstract

Kyphoscoliotic Ehlers–Danlos syndrome (kEDS) is a rare autosomal connective tissue disorder characterized by progressive kyphoscoliosis, congenital muscular hypotonia, marked joint hypermobility, and severe skin hyperextensibility and fragility. Deficiency of lysyl hydroxylase due to variants of the *PLOD1* gene has been identified as a pathogenic cause of the disease. Vascular fragility in kEDS has rarely been reported. Here, we report a 15-year-old Chinese boy with kEDS-*PLOD1* who presented with a superior mesenteric aneurysm and severe vascular complications. The patient underwent emergency hybrid surgery combining hemostasis by laparotomy and stent graft placement superior to the bleeding artery by endovascular intervention. The patient's presentation improved postoperatively. Unfortunately, the patient died despite medical intervention. Whole exome sequencing identified compound heterozygous variants in the patient's *PLOD1* gene: a reported variant, c.1095C > T, and a novel variant, c.1262delC. The c.1262delC variant is a frameshift variant that results in a premature stop codon and loss of gene function. Overall, this case report further expands the genetic landscape of kEDS and suggests that vascular intervention in these patients requires individualized assessment of vessel function and local perfusion status.

## 1 Introduction

Ehlers–Danlos syndrome (EDS) is a rare autosomal connective tissue disorder with hereditary heterogeneity. EDS is currently divided into 13 subtypes based on key characteristics, including joint activity, excessive skin stretching, and tissue fragility. The clinical manifestations of EDS subtypes are different (1, 2). Some subtypes present with skeletal muscle systems and rupture of blood vessels and cavity organs, leading to disability or death in severe cases (3). Kyphoscoliotic EDS (kEDS; OMIM 225400, previously EDS type VIA) is a rare autosomal recessive connective tissue disorder (4). However, the prevalence of kEDS remains unclear. The estimated incidence is 1:100,000 live births with a carrier frequency of 1:150 (5). kEDS was originally defined at the biochemical level, based on a family study in which two sisters developed progressive scoliosis, joint laxity, recurrent joint dislocations, and microcorneal and ocular tissue fragility. The identified cause was a deficiency of 2-ketoglutarate 5- dioxygenase 1 [*PLOD*1 or lysyl hydroxylase 1 (LH1)] caused by *PLOD*1 mutation, a specific form of EDS kyphosis. LH1 deficiency leads to insufficient hydroxylation of collagen lysyl residues and glycosylation of hydroxylysyl residues in the Xaa-Lys-Gly collagen chain, which leads to damage to collagen crosslinking and mechanical instability of the affected connective tissues (6). More than 40 different mutations in *PLOD*1 have been identified in kEDS (7). *PLOD*1 mutations have rarely been reported in the Chinese population. It has been reported that the risk of complications, such as spontaneous aneurysm or rupture of medium-to-large arteries, in kEDS-*PLOD*1 patients increases significantly with age. Here, we report the case of a 15-year-old boy with kEDS caused by compound heterozygous variants in *PLOD*1, who suffered with fatal vascular defects.

## 2 Methods

### 2.1 Research subjects

A Chinese family (three members) with kEDS-PLOD1 was recruited from Xinhua Hospital affiliated with the Shanghai Jiaotong University School of Medicine. This study was approved by the Ethics and Human Research Committee of Xinhua Hospital (Approval No. XHEC-D-2023-235).

### 2.2 Clinical description

A 15-year-old boy was admitted to the hospital with a low-grade fever, abdominal pain, nausea, constipation, and fatigue for three days. Two hours prior to admission, the child presented with seizures that lasted for up to 5 min. The patient was diagnosed with epilepsy and kEDS-*PLOD*1 at the age of 12 and 14 years of age, respectively. Antibiotics were administered before admission. Family history of cardiovascular diseases was denied.

### 2.3 Genetic screening

Genomic DNA was extracted from the peripheral blood samples of the family members and whole exome sequencing of the proband was performed to screen the pathogenic variants. Briefly, genomic DNA was fragmented to an average size of 150 bp using a S220 Focused-ultrasonicator (Covaris, Massachusetts, USA). A DNA Sample Prep Reagent Set (MyGenostics, Beijing, China) was used to prepare standard Illumina libraries. Amplified DNA was captured using a GenCap capture kit (MyGenostics) according to the manufacturer's instructions. The enrichment libraries were sequenced on an Illumina HiSeq X Ten sequencer for paired reading (150 bp). Variant calling, annotation, and prioritization were performed using the MyGenostics Cloud platform. Variants validation and co-segregation with the phenotype in the family were achieved by PCR and Sanger sequencing. Variant nomenclature was described according to the *PLOD*1 transcript reference NM_000302.4, following the Human Genome Variation Society (HGVS) guidelines.

### 2.4 Quantitative real-time PCR (qRT-PCR)

Total RNA was extracted from fresh blood samples using a standard procedure with TRIzol (Invitrogen; CA, USA) and chloroform. Reverse transcription was performed immediately after RNA extraction using PrimeScript RT Master Mix (TaKaRa; Kusatsu, Japan). Real-time qPCR was carried out using a QuantStudio Dx Real-Time instrument (Thermo Fisher; NY, USA) with the primer pair PLOD1-qF (5′- GAGGTGCGGATGGCGAAT-3′) and PLOD1-qR (5′-TCGCCACTCTTGCCACCAGC-3′). PLOD1 mRNA expression levels were normalized to ACTB (ACTB-qF: 5′-GCACAGAGCCTCGCCTT-3′ and ACTB-qR: 5′-GTTGTCGACGACGAGCG-3′), a housekeeping gene, and presented as fold changes. Each reaction was carried out in three technical replicates.

## 3 Results

### 3.1 Clinical process

**Figure 1:**
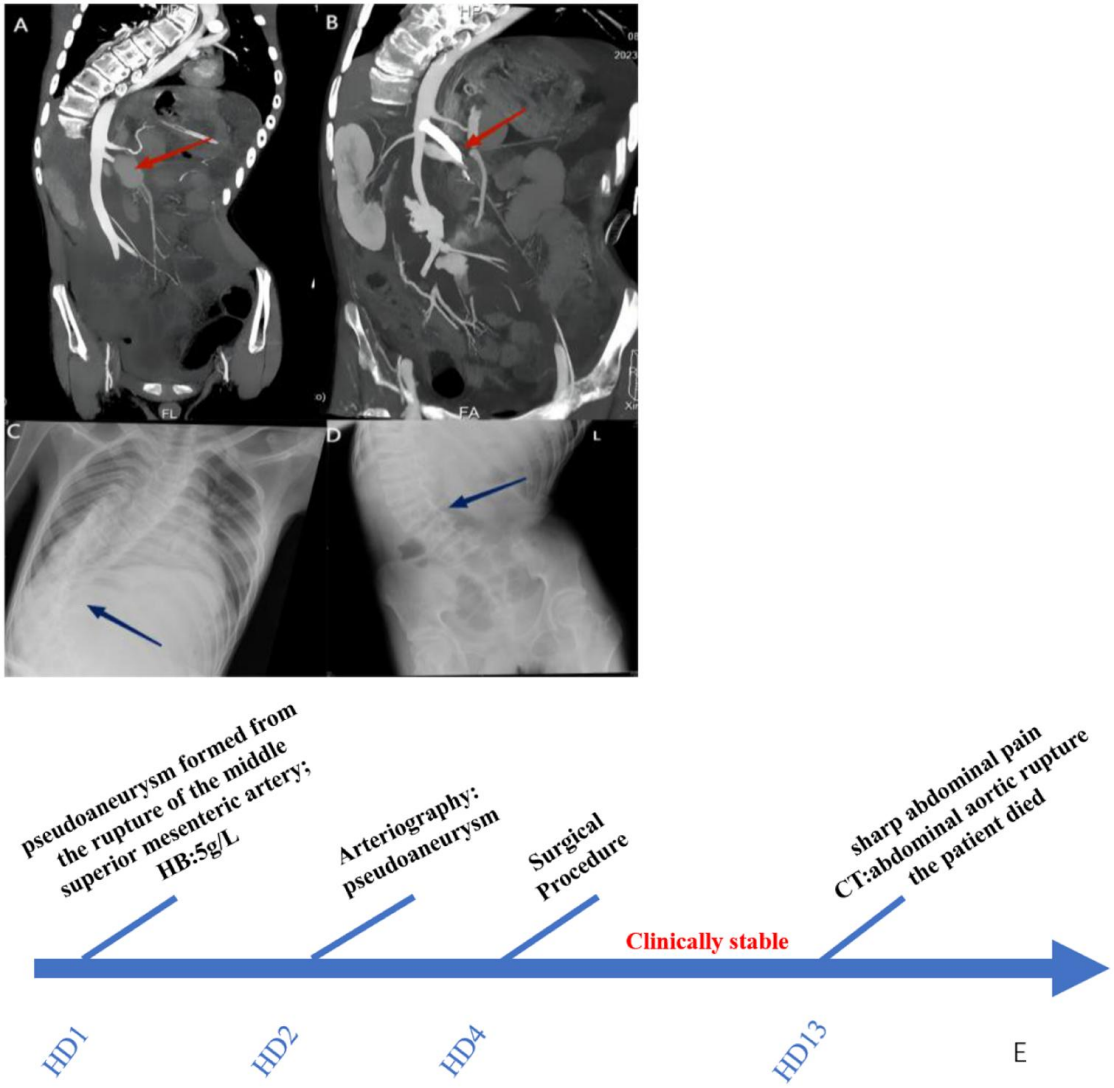
Clinical imaging of the patient. **(A)** The red arrow indicates the superior mesentery artery pseudoaneurysm. **(B)** The blue arrow indicates abdominal aortic rupture. **(C,D)** The blue arrow indicates kyphoscoliosis. **(E)** Timeline of Care.

After admission, compared with the patient's baseline level, complete blood test showed a decreased hemoglobin level of 5 g/L, while white blood cell and platelet counts were normal. Fecal occult blood test results were negative. Computed tomography (CT) scans of the head and thorax were normal, except for scoliosis. Contrast-enhanced abdominal CT revealed a pseudoaneurysm formed from the rupture of the middle superior mesenteric artery (Figure 1A). The child underwent arteriography, which identified vascular lesions at a small branch of the superior mesenteric artery and a pseudoaneurysm formed around the bleeding site. The patient experienced rupture of the branch artery, which was related to damage to the collagen crosslinking of the vessels, the main defect of patients with kEDS. Bleeding temporarily stopped when the pseudoaneurysm formed. The patient underwent emergency hybrid surgery combining hemostasis by laparotomy with stent graft placement superior to the bleeding artery by endovascular intervention. The patient's presentation improved postoperatively. Unfortunately, 10 days later, sharp abdominal pain developed, and a contrast CT scan revealed an abdominal aortic rupture, resulting in intraabdominal hemorrhage with profuse hemoperitoneum (Figure 1B). The patient immediately developed hemorrhagic shock, and his condition deteriorated rapidly. Several hours later, the patient died, despite medical intervention (Figure 1E).

### 3.2 Compound heterozygous *PLOD*1 variants co-segregate with the disease phenotype

**Figure 2:**
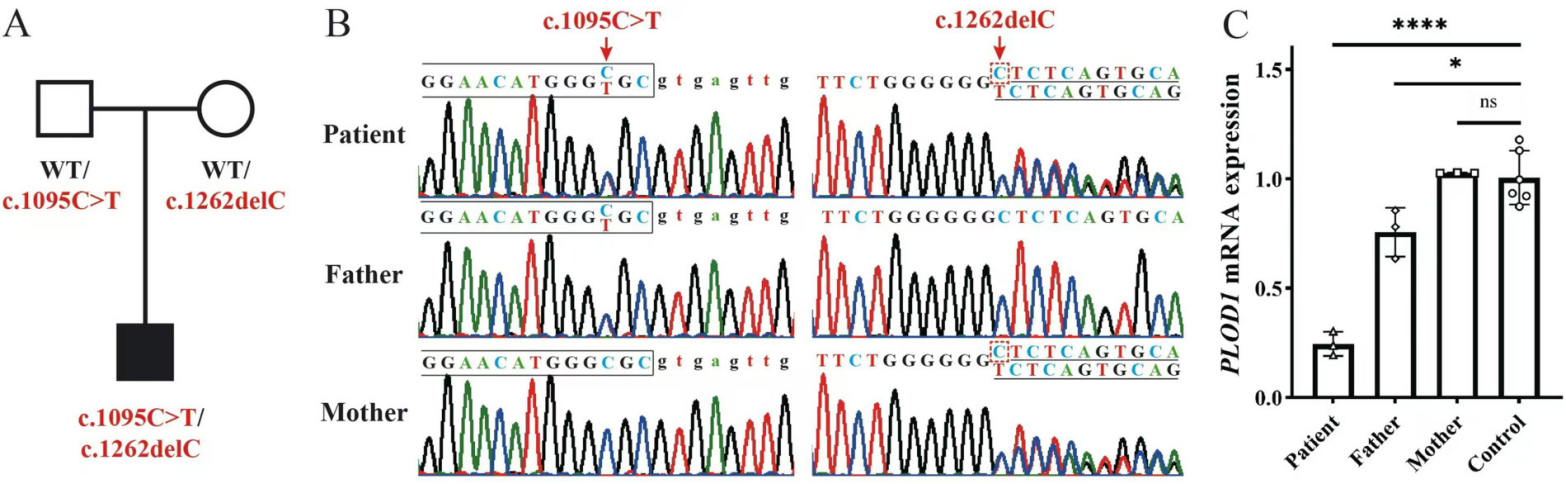
Compound heterozygous *PLOD*1 variants co-segregate with the disease phenotype. **(A)** Detection and certification of the variant. **(B)** Pedigree chart. **(C)** Comparison of familial *PLOD*1 expression.

In this patient, compound heterozygous variants were found in *PLOD*1 (Figures 2A,B). The c.1095C > T variant was a recurrently reported synonymous variant that functionally generates a new active splice site, resulting in abnormal mRNA splicing and loss of gene function (8, 9). The other variant, c.1262delC, has not been reported before. This frameshift variant was predicted to cause a premature stop codon and loss of *PLOD*1 function. Real-time qPCR analysis showed that the *PLOD*1 expression level in the peripheral blood of the patient was significantly decreased compared to those in the parents and healthy control (Figure 2C). These results indicate that both variants resulted in abnormal transcripts with a premature stop codon and eventually caused loss of *PLOD*1 function through nonsense-mediated mRNA degradation (NMD).

## 4 Discussion

EDS is a rare genetic disorder of the connective tissue. Patients often present varying degrees of skin hyperextension, joint hypermobility, and excessive tissue fragility. kEDS (OMIM 225400, previously EDS type VIA) is a rare autosomal recessive connective tissue disorder. It is characterized by congenital muscle hypotonia, congenital or early onset progressive kyphoscoliosis, generalized joint hypermobility, skin and scleral fragility, facial dysmorphia, and vascular fragility (2, 8). kEDS-*PLOD*1 (OMIM 225400) results from LH1 deficiency due to a pathogenic variant of the *PLOD*1 (procollagen-lysine, 2-oxoglutarate 5-dioxygenase 1) gene. LH1, encoded by the *PLOD*1 gene, specifically hydroxylates helical lysyl residues in Xaa-Lys-Gly collagen sequences to hydroxylysyl residues. The latter serve as attachment sites for carbohydrate units (galactose or glucosylgalactose) (10). The LH1 activity of fibroblasts is lower in patients with kEDS-*PLOD*1 compared with healthy individuals. Studies have confirmed the degeneration of collagen fibers and abnormalities in smooth muscle cells in living mouse tissues, indicating the occurrence of aortic dissection in mice, which leads to aging and rupture of vascular function (11). Vascular lesions can occur in the arterial or venous system (5, 12–14). Severe and life-threatening complications, such as spontaneous aneurysms or spontaneous ruptures of medium-to-large arteries, have been reported in patients affected by kEDS*-PLOD*1 (13, 14). Although neonatal cerebral hemorrhage has been reported (15–19), vascular incidents usually occur during the transition from youth to adulthood (16, 20). Patients with kEDS-*PLOD*1 are at risk of developing severe vascular complications at any age (14). According to previous reports, patients can develop non-vascular complications such as recurrent pneumonia and heart failure (caused by severe kyphotic scoliosis), chronic respiratory failure, glaucoma, retinal detachment, as well as vascular complications such as arterial rupture, aneurysm, coronary aneurysm, aneurysm rupture, intracranial hemorrhage due to intracranial vascular lesions, and vascular complications of the circulatory system, which are more likely to be life-threatening (14, 21). After diagnosis, these patients should be offered cardiovascular management such as regular cardiovascular follow-up. Currently, the only recommended imaging screening for patients with EDS is an echocardiogram to investigate the valves and coronary and aortic roots (22). Regular screening with abdominal vascular and cardiac ultrasound may be beneficial in these patients as well. Blood pressure monitoring and early beta-blocker therapy are recommended to prevent arterial complications. Surgical interventions should be advised to avoid complications such as vessel rupture (13). In the present case, the intraabdominal vascular lesion of the superior mesenteric artery was affected, and a pseudoaneurysm formed after the vessel broke. Our treatment failure may have been related to vascular fragility and disturbance of the abdominal circulation after stent placement. Careful assessment of vessel function and reperfusion status should be performed in patients with decompensated symptoms.

In previous reports of *PLOD*1 variants, the most common was a large duplication of 10–16 exons that was found in >20% of patients with kEDS-*PLOD*1, and the others were point variants, insertions, and deletions that caused premature termination of codons and splicing variants that caused exon skipping (8). In the present case, two variants were found: c.1095C > T and c.1262delC. The c.1095C > T variant is previously known, whereas the c.1262delC variant is novel. The c.1095C > T variant was previously reported in China (9). The c.1095C > T synonymous variant in *PLOD*1 introduces a single-nucleotide transition from C to T at nucleotide 1095, which generates a new active splice site, resulting in abnormal mRNA splicing and loss of gene function. This proved that the c.1095C > T variant was the cause of the premature termination codon in exon 105. The other variant c.1262delC has not been reported before. This frameshift variant leads to a premature stop codon and loss of gene function. This frameshift variant was predicted to cause a premature stop codon and loss of *PLOD*1 function. We identified one novel variant of *PLOD1*, extending the mutation spectrum of *PLOD1*. Reat-time qPCR analysis showed that *PLOD*1 expression levels in the peripheral blood of the patient were significantly lower compared to those in the parents and healthy control. The qPCR results suggest that the abnormal transcripts caused by the two variants led to mRNA instability. This defect may lead to the loss of gene function via NMD. Molecular genetic testing revealed that these two variants were related to kEDS-*PLOD*1.

## 5 Conclusion

Patients with kEDS-*PLOD1* are at risk of abdominal vascular fragility and anterior aneurysm rupture. Vascular intervention in these patients requires individualized assessment of vessel function and local perfusion status. Stent graft placement and surgical repair may be options after careful assessment. 1095C > T and c.1262delC mutations were found in this case. The c.1095C > T variant is previously known, whereas the c.1262delC variant is novel.

## Data Availability

The datasets presented in this article are not readily available because of ethical and privacy restrictions. Requests to access the datasets should be directed to the corresponding author/s.
